# High-intensity interval training improves the reactive strength index and motor ability of youth football players

**DOI:** 10.1186/s13102-026-01560-9

**Published:** 2026-02-02

**Authors:** Serkan Kızılca, Muhammed Zahit Kahraman, Sedat Okut, Ersin Arslan, Ömer Faruk Bilici, İsmail Çelik, Tayfun İşlen, Sebahattin Altıntaş, Muhammed Fatih Bilici

**Affiliations:** 1 Faculty of Sports Sciences, Bitlis Eren University, Bitlis, Turkey; 2 Faculty of Sports Sciences, Muş Alparslan University, Muş, Turkey; 3 Faculty of Sports Sciences, Van Yüzüncü Yıl University, Van, Turkey; 4 Institute of Health Sciences, Marmara University, İstanbul, Turkey; 5 Institute of Social Sciences, Muş Alparslan University, Muş, Turkey; 6 Winter Sports and Sports Sciences Institute, Atatürk University, Erzurum, Turkey

**Keywords:** Adolescents, Motor performance, Training adaptation

## Abstract

**Background:**

The aim of this randomized controlled trial was to determine the effects of a four-week running-based high-intensity interval training (HIIT) intervention on the reactive strength index (RSI) and motor abilities in youth football players. In this context, the contributions of the running-based HIIT protocol HIIT protocol, implemented in addition to regular football training, were evaluated on performance indicators such as speed, back strength, change of direction (COD), and countermovement jump (CMJ). This study aims to contribute to the development of scientifically grounded training programs to support performance improvement in youth football players.

**Methods:**

A quantitative research design was employed in this study, specifically a randomized pretest–posttest controlled experimental design. Twenty male youth football players, aged 11–13 years, who voluntarily participated in the study were randomly allocated to either the experimental group (EG; *n* = 10) or the control group (CON; *n* = 10). The EG participated in a running-based HIIT program three times per week for four weeks by replacing a low-to-moderate-intensity technical–tactical segment of their regular football training, whereas the CON continued with only regular football training during this period without any additional training volume. The players underwent RSI, 10 m, 20 m, and 30 m sprint tests, back strength tests, the Illinois Agility Test, and CMJ tests both before the training sessions and at the end of the four-week intervention.

**Results:**

According to the repeated-measures ANOVA results, pretest values did not differ significantly between the groups (*p* > .05). Over the 4-week period, significant improvements with large effect sizes were observed in RSI, sprint performance (10, 20, 30 m), back strength, COD, and CMJ tests (*p* < .05; ηp² = 0.29–0.55). Significant group × time interactions were also detected for all variables, again with large effect sizes (*p* < .05; ηp² = 0.20–0.30), indicating greater improvements in the EG compared to the CON. However, despite these favourable within-group changes, between-group post-test comparisons did not reach statistical significance.

**Conclusions:**

This study revealed that a 4-week HIIT intervention applied to youth male football players had positive effects on the RSI and motor abilities, as evidenced by large within-group improvements and significant group × time interactions, despite the absence of statistically significant between-group post-test differences. These findings suggest that HIIT protocols should be integrated into training programs as an effective strategy to improve explosive strength, COD, and overall functional performance in young athletes.

**Trial registration:**

This randomized controlled trial was retrospectively registered with ISRCTN (ISRCTN45188963) on 16 July 2025 due to administrative delays during ethics approval and registry processing.

**Supplementary Information:**

The online version contains supplementary material available at 10.1186/s13102-026-01560-9.

## Introduction

It is highly important for football players to possess a high level of physical capacity [1]. Football, as an intermittent sport with varying energy demands, requires players to sustain prolonged exertion while performing powerful movements [2]. Recent studies highlight the importance of training methods in developing young football players, with High-Intensity Interval Training (HIIT) emerging as an effective approach to improve both aerobic and anaerobic performance [3–5]. For example, Bibić et al. [6] reported that HIIT protocols with change-of-direction significantly enhanced sprint and agility outcomes in young football players, and Panascì et al. [7] showed that a 4-week HIIT program improved aerobic fitness and sprint endurance in adolescent football players, supporting the applicability of HIIT-based interventions in youth populations. Also, it has been demonstrated that HIIT is highly effective in improving fundamental motor abilities such as sprint speed, change of direction (COD), and endurance [8, 9].

The reactive strength index (RSI), defined as the ratio of jump height to ground contact time, is a valid indicator of explosive strength and rapid force production [10, 11]. It has been shown to be strongly associated with sprinting and COD performance in youth football players [12, 13]. In football, where sudden accelerations, decelerations, and directional changes are essential, RSI is considered a key determinant of athletic performance [14]. As a high-intensity intermittent sport, football particularly benefits from HIIT, which can improve both RSI and other biomotor abilities [15, 16]. Moreover, the development of motor abilities in youth athletes is linked to growth and maturation, and RSI plays an important role in enabling effective responses to dynamic game situations [17]. Early adolescence (ages 11–13) is widely regarded as a sensitive developmental phase marked by accelerated neuromuscular maturation, heightened responsiveness of the stretch–shortening cycle, and increased trainability of explosive motor actions [18]. Despite the growing body of literature examining HIIT-induced performance adaptations in adolescent and adult football players, evidence specifically addressing RSI responses to HIIT in early adolescent male football players remains scarce [19]. Most existing studies have focused on older adolescents (U17–U19) or adult populations, thereby limiting insight into age-specific neuromuscular adaptations to HIIT during early adolescence [20]. Therefore, investigating RSI responses to HIIT in this specific age group is of particular scientific relevance.

Evidence indicates that HIIT significantly enhances sprint ability and COD performance in youth football players [19, 21]. Recent studies have also demonstrated that HIIT can be effective in improving different performance components in young football players [12]. It has been reported that concurrent high-intensity and strength training during the pre-season enhanced muscle power and aerobic performance, while two different short in-season HIIT formats led to significant gains in aerobic capacity and neuromuscular performance [22, 23]. These findings highlight the potential of HIIT protocols to improve sprinting, jumping, COD and overall physical performance, and underscore the need for further research in youth football players. Furthermore, HIIT interventions have been found to increase countermovement jump (CMJ) and RSI, reflecting gains in explosive strength and stretch shortening cycle efficiency [24, 25]. Although HIIT has been shown to influence various neuromuscular outcomes, the literature contains very limited evidence regarding its potential impact on back strength—a variable in which the present study observed a very large effect size. Highlighting this outcome may provide a novel contribution to HIIT research in youth athletes. In addition, HIIT has been associated with improvements in muscular strength, including trunk and back strength, through neuromuscular adaptations [26]. Considering that the 11–13 age range corresponds to a critical developmental window characterized by rapid growth, restructuring of motor coordination, and heightened neuromuscular plasticity around the period of peak height velocity [27], understanding the responsiveness of this age group to HIIT is particularly important. Overall, these findings suggest that HIIT is not only effective for aerobic and anaerobic capacity but also provides direct benefits for RSI, sprint, COD, CMJ, and muscular strength key performance outcomes assessed in this research [19, 21, 24–26].

The primary aim of this study was to examine the effects of HIIT on RSI and biomotor abilities (sprint, strength, COD, and CMJ) in 11–13-year-old male football players. While HIIT has been studied in youth football, the specific effects of HIIT on RSI in early adolescent (11–13 years) male players remain underexplored, which constitutes a primary focus of the present study. Additionally, the study’s findings regarding back strength may offer new insights, as this variable has received little attention in previous HIIT research. In line with this purpose, we hypothesised that a 4-week running-based HIIT program would result in significant improvements in RSI, sprint performance, strength, COD, and CMJ within the experimental group. Given the sample size considerations, between-group differences were considered exploratory rather than confirmatory. By highlighting the potential of HIIT to enhance RSI and motor abilities, this research seeks to contribute to the scientific optimization of training programs for young football players and to provide practical guidance for coaches and educators.

## Methods

**Fig. 1:**
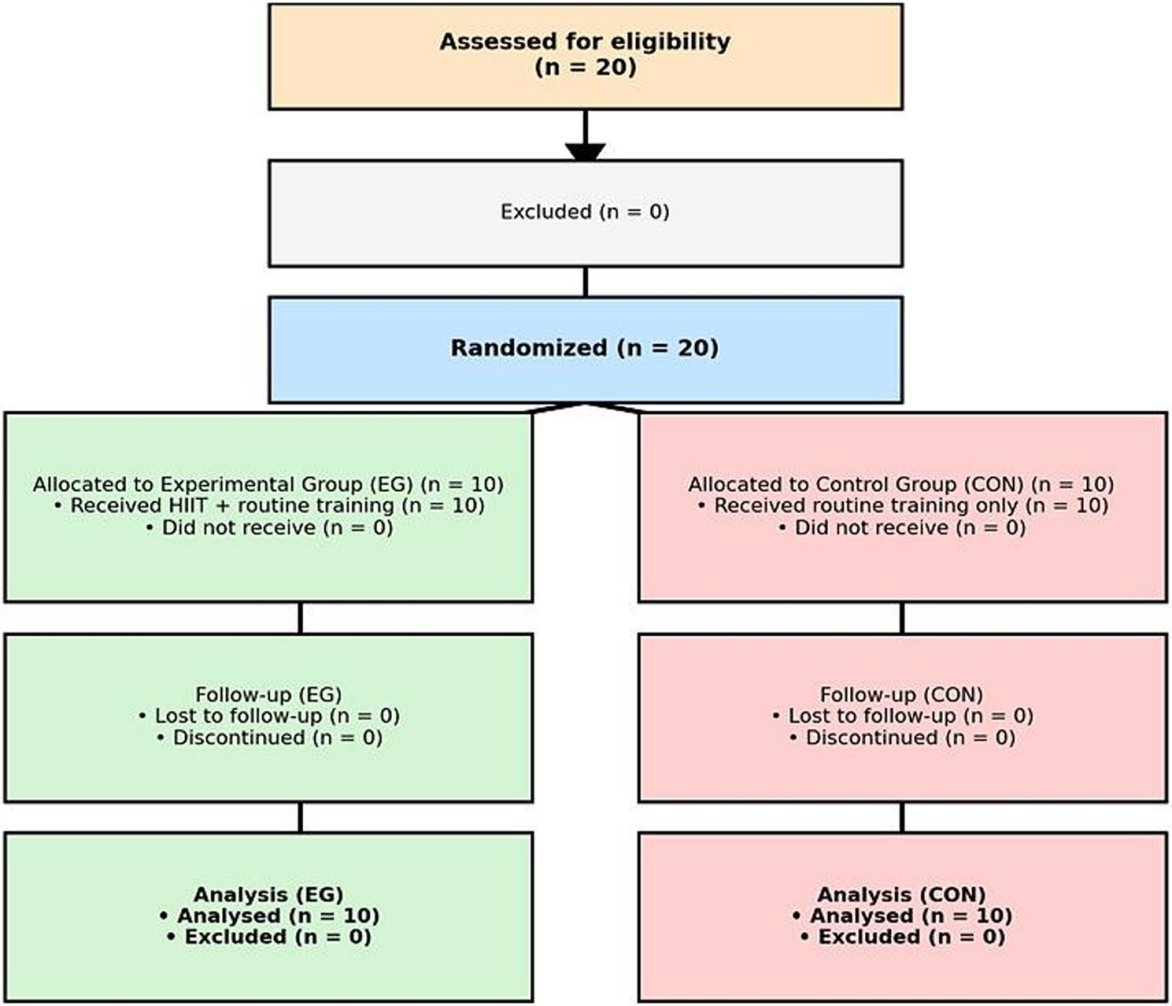
CONSORT flow diagram of participants. The diagram shows the number of participants assessed for eligibility (*n* = 20), randomized into experimental (EG, *n* = 10) and control (CON, *n* = 10) groups, followed up, and analyzed. Importantly, no participants were lost to follow-up or excluded from the analysis, ensuring complete retention throughout the study

This study employed a randomized controlled pre–post test design, and was conducted in accordance with the CONSORT. Participants were recruited from a local football academy via coach announcements and informational leaflets distributed to players and parents one week before enrolment. Written informed consent was obtained from all players and their legal guardians, and assent was obtained from the child participants prior to participation. The participants were randomly allocated into the Experimental Group (EG; *n* = 10) and the Control Group (CON; *n* = 10) using computer-assisted block randomisation in Microsoft Excel (block size: 4). The allocation sequence was concealed and not accessible to outcome assessors. All pre- and post-intervention evaluations were conducted by assessors blinded to group allocation (Fig. 1).

**Fig. 2:**
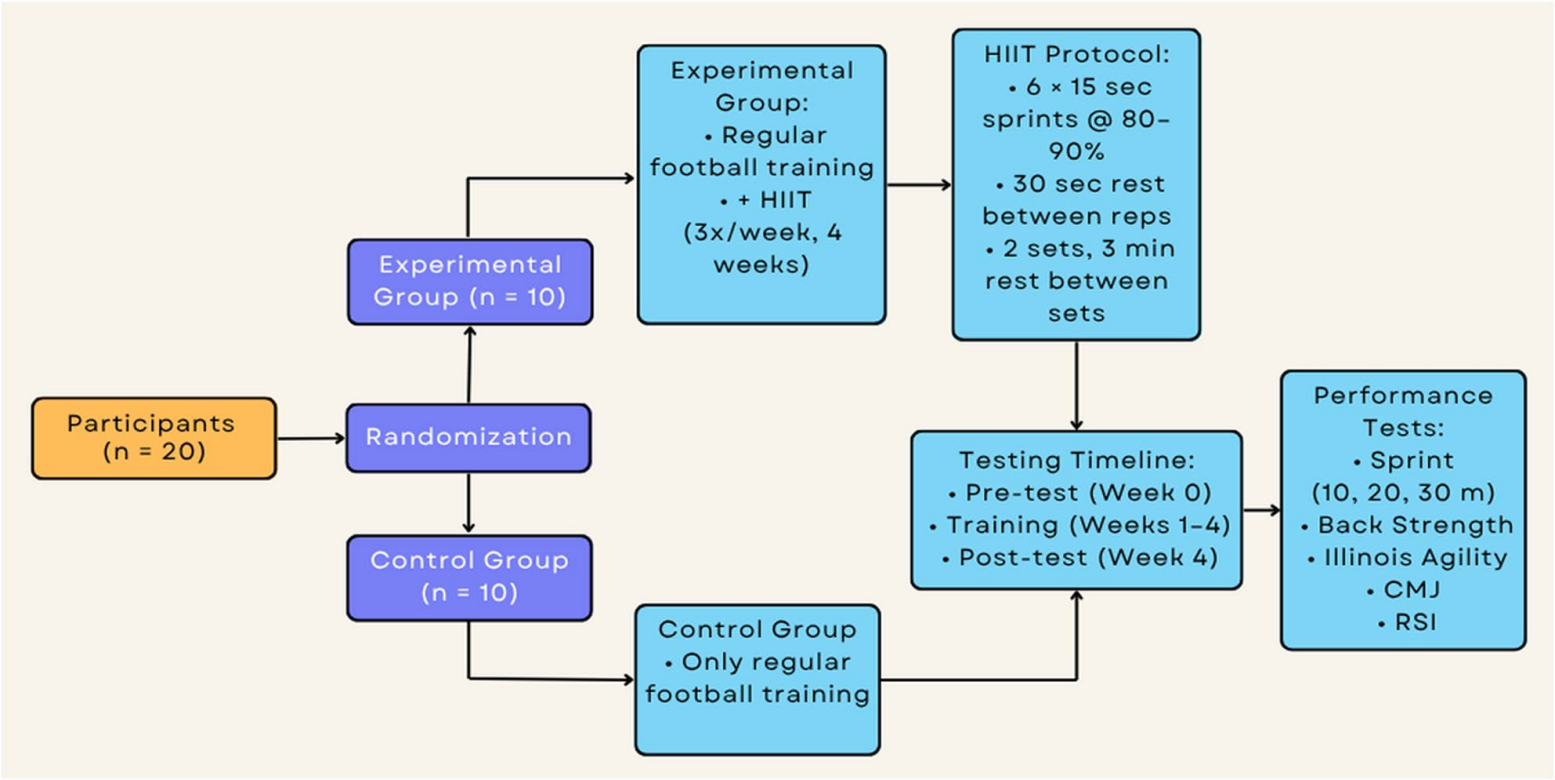
Schematic representation of the study design. The figure shows the randomization of participants into the experimental group (EG) and control group (CON), the 4-week HIIT intervention added to routine football training for the EG, and the timing of pre- and post-test performance assessments

The EG group performed running-based HIIT three times a week for four weeks, in addition to their regular football training. The CON group, on the other hand, continued with their routine football training, which consisted of three sessions per week (Monday, Wednesday, Friday) lasting 75–90 min each. Each session included a standardized warm-up (10–15 min), technical and tactical exercises (40–50 min), and small-sided games or match play (20–25 min). In the EG, approximately 8 min of HIIT drills were incorporated by replacing a portion of the technical-tactical activities, so that the total training session duration was matched between groups. No additional conditioning or HIIT sessions were performed. One week before data collection, all participants were provided with detailed information about the test protocols, and they were provided with instructional videos explaining the test procedures. Pre- and post-tests were conducted to evaluate performance parameters including RSI, sprint (10, 20, 30 m), back strength, COD, and CMJ. It was ensured that the athletes were informed about every stage of the study (Fig. 2).

### Participants

This study included a total of 20 male youth football players from a football academy (mean age: 12.15 ± 0.81 years; height: 152.65 ± 8.08 cm; weight: 41.02 ± 8.48 kg). All players registered in the relevant age category (11–13 years) at the academy were assessed for eligibility and invited to participate in the study. Of the eligible players, 20 agreed to participate and completed all testing procedures, resulting in a participation rate of 100%. No eligible players declined participation or were excluded after enrolment. The required sample size was determined using G*Power software (version 3.1.9.4). Previous interventions with youth football players have reported medium-to-large effects on physical performance outcomes. For instance, Ferrete et al. [28] observed large effects (Cohen’s d ≈ 0.80) on CMJ and sprint performance in prepubertal players following combined strength and HIIT. Similarly, Sperlich et al. [29] reported medium-to-large partial eta squared values (ηp² ≈ 0.12–0.14) for aerobic capacity and sprint performance improvements in 14-year-old football players after HIIT. Moreover, Ramirez-Campillo et al. [30] and Michailidis et al. [31] demonstrated medium-to-large effects (d ≈ 0.70–0.90) on RSI, sprint, and COD performance in young football players. Based on these findings, a large effect size (Cohen’s f = 0.40; equivalent to d ≈ 0.80; ηp² ≈ 0.14) was adopted for the a priori power analysis (test family: F tests; ANOVA repeated measures, between–within interaction; groups = 2; measurements = 2; assumed correlation among repeated measures = 0.50; ε = 1). The analysis indicated that a total sample of 20 participants (10 per group) would be sufficient to achieve 80% power at α = 0.05.

Prior to the commencement of the study, detailed information regarding the research was provided to the youth participants and their parents. Initially, all the participants were informed that the study was conducted on a voluntary basis and that they would only be included if they agreed to participate. A voluntary assent form was signed by the youth participants who agreed to take part in the study, and parental consent was obtained from their legal guardians. The inclusion criteria for the study were being no older than 13 years, providing written consent, and obtaining parental consent. The exclusion criteria included the presence of chronic diseases, orthopedic disorders, infections, the necessity of continuous medication use, and the decision to withdraw from the study at any stage. Detailed information regarding the training protocol and measurement procedures was provided to both the youth participants and their parents. Participants who met all the inclusion criteria and their families were instructed to maintain a healthy diet and regular sleep patterns. Additionally, participants were advised to avoid intense physical activity, caffeine consumption, and the use of medication or dietary supplements in the 24 h preceding the measurements. Exclusion criteria included the presence of any known chronic disease, neurological condition, or musculoskeletal or orthopaedic disorder that could affect physical performance or participation in high-intensity exercise. Eligibility was assessed through parental self-report and a standard health questionnaire completed prior to enrolment. All participants were members of a football academy and were routinely monitored through the academy’s coaching and health monitoring procedures; no player was excluded due to medical or orthopaedic conditions. The study was conducted in accordance with the Declaration of Helsinki (https://sites.jamanetwork.com/research-ethics/index.html). Prior to the study, ethical approval was obtained from the Non-Invasive Clinical Research Ethics Committee of Bitlis Eren University, with decision number 13, dated 02.01.2025, confirming the study’s compliance with ethical guidelines.

### Trial registration

The trial was registered retrospectively with ISRCTN (ISRCTN45188963) due to administrative and procedural timelines related to ethics committee approval and registry processing; however, ethical approval and written informed consent from participants and their parents were obtained prior to participant enrollment and the commencement of the intervention.

### Study setting

The trial was conducted in an outdoor community sports setting at the Tatvan Fairground in Bitlis, Turkey. All HIIT sessions and physical performance assessments were performed on a natural grass football field located at this site, prior to regular football training. All testing and training sessions were conducted at 4:00 p.m. to ensure consistency in environmental and physiological conditions across participants.

### Training protocol and implementation

The HIIT protocol applied to the athletes was conducted for four weeks, three days per week (Monday, Wednesday, and Friday). Before starting the training sessions, the participants were informed about the training protocol and its implementation process. Additionally, the potential risks and benefits of the training were explained to them. No harms or adverse events occurred during the trial. Participants were monitored throughout the intervention sessions. Warm-up and cooldown phases were included in every session to minimize injury risk, and all activities were supervised by trained staff.

The HIIT program was implemented for the youth football players in the EG in addition to their routine football training (Table 1). Each session began with a standardized 15-minute warm-up consisting of slow-paced running (7 min), stretching (4 min), and jumping exercises (4 min) to minimize the risk of injury. The training protocol consisted of six 15-second running repetitions performed in two sets. The running bouts were executed at individualized speeds that corresponded to the weekly prescribed training intensities (80%, 85%, and 90%). For this reason, no fixed running distance was prescribed; each repetition was completed at a speed that allowed the player to reach the required intensity, ensuring individualized loading. Running speed was individualised according to the heart rate–based intensity criteria described in the “Determination of training intensity” section. In the EG, the 8-minute HIIT block was performed during the same segment of the session in which the CON group completed low-to-moderate-intensity technical–tactical drills (e.g., passing patterns, positional shadow play, and simple possession games). Thus, the HIIT block replaced an equivalent duration of routine technical–tactical work rather than being added as extra training. Both groups completed the sessions in the same total duration, ensuring that the overall training time was equalized between EG and CON. Although total session duration and weekly training frequency were matched between groups, total weekly training load (including football training) was not quantitatively monitored using internal or external load metrics. Therefore, load equivalence beyond session duration cannot be fully confirmed. Sessions were scheduled three times per week (Monday, Wednesday, Friday) over a four-week period, with at least 48 h of recovery between sessions to ensure optimal physical adaptation.

All sessions were delivered and closely supervised by certified sports science researchers and experienced coaches trained in HIIT methodologies. Parental observation was permitted during the sessions. No deviations from the planned intervention occurred, and all participants in the EG fully adhered to the protocol by attending all 10 sessions (10/10). In terms of concurrent care, both groups continued with their usual team-related activities outside the study sessions, and no additional structured physical or cognitive training programs were introduced. Both groups were advised not to engage in any additional structured physical or cognitive training programs, and the use of ergogenic aids or supplements was not permitted during the intervention period.

### Safety and harms

All training sessions were supervised by certified personnel, and standardized warm-up and cooldown routines were applied in every session to minimize injury risk. Throughout the intervention period, no adverse events, injuries, or unintended effects were observed in either group. All participants completed the intervention safely under professional supervision.

### Determination of training intensity

Target heart rate (THR) zones were calculated using the Karvonen formula [32]: THR = [(HR_max − Resting HR) × intensity] + Resting HR. The Tanaka model [33] was used to estimate HR_max, which is considered more accurate than the traditional “220 − age” formula for younger populations, and is defined as HR_max = 208 − (0.7 × age). For players aged 11–13 years, HR_max was approximately 200 bpm; based on this value, the target training zones corresponded to 160 bpm for 80% intensity, 170 bpm for 85%, and 180 bpm for 90%.

During all HIIT sessions, heart rate was continuously monitored with chest-strap telemetry devices (Polar^®^, Kempele, Finland). Coaches provided real-time feedback to ensure that each player remained within their individually prescribed THR zones (Table 1). If an athlete’s HR exceeded the zone, the running pace was reduced; if it fell below the zone, intensity was increased. This individualized adjustment allowed participants to consistently perform within the intended intensity range across all sessions.

**Table 1:** High-intensity interval training protocol

Training Variables	Week 1	Week 2	Week 3	Week 4
Training Intensity (%)	%80	%85	%85	%90
Exercise Duration (sec)	15 s	15 s	15 s	15 s
Number of Repetitions	6	6	6	6
Rest Between Repetitions (sec)	30 s	30 s	30 s	30 s
Number of Sets	2	2	2	2
Rest Between Sets (min)	3 min	3 min	3 min	3 min
Theoretical Target HR Zone (bpm)	160	170	170	180
Total Training Duration (min)	8 min	8 min	8 min	8 min
Work-to-Rest Ratio	1:2	1:2	1:2	1:2
Training Frequency (days/week)	3 days	3 days	3 days	3 days

### Test procedures

All physical performance assessments of the participants were conducted on a natural grass football field. Before testing, participants’ body weights were measured using an electronic scale (Tanita BC-418 MA, Japan) with a precision of 0.1 kg, and heights were measured using a stadiometer (SECA, Germany) with a precision of 0.01 m. Prior to testing, all participants completed a standardized 15-minute warm-up protocol, consisting of slow-paced running (7 min), stretching exercises (4 min), and jumping drills (4 min). To ensure data reliability, the tests were distributed over two separate days, with a 48-hour rest period between sessions to ensure full recovery. On the first day, sprint tests (10 m, 20 m, and 30 m) and back strength tests were performed; on the second day, RSI, CMJ, and Illinois agility tests were administered.

### Reactive strength index (RSI) measurement

RSI measurements were conducted using the Smart Speed Lite Smart Jump system (Fusion Sport, AU). Before each trial, the system was reset. Participants initiated the assessment by stepping forward from a 30-cm box, performing a single drop jump. This initial drop jump was used solely to activate the stretch–shortening cycle and was not included in the analysis. Immediately after landing from the initial drop jump, with no additional rest interval, participants performed 10 consecutive rebound jumps on the contact mat. During all jumps, participants kept their hands placed on their hips and were instructed to “jump as high as possible while minimizing ground contact time.” Vertical ground reaction force data were collected at a sampling rate of 1000 Hz over a 15-second period. RSI was calculated individually for each of the 10 rebound jumps, and the highest five RSI values were averaged and used for analysis. This procedure corresponds directly to the widely used 5/10 repeated-jump RSI protocol described in the literature. Compared to single drop-jump assessments, the repeated-jump approach offers improved measurement reliability, reduces single-trial variability, and better reflects the repeated stretch–shortening cycle demands commonly observed in football. Therefore, the present study adopted this multi-jump protocol, consistent with previous research evaluating lower-limb reactive performance under dynamic or fatigue-like conditions [34–37]. The test was performed twice, with a 90-second rest interval between trials, and the highest mean RSI value from the two trials was used for analysis. RSI was calculated using the standard formula [38]: RSI = Jump Height (mm)/Ground Contact Time (ms).

### Speed tests (10 m, 20 m, and 30 m)

A 10-, 20-, and 30-m sprint test was performed using the Smart Speed Lite electronic timing system (Fusion Sport, AU), with timing gates placed at the start, 10 m, 20 m, and finish line. Participants started from a standing position 50 cm behind the line. Each sprint was performed twice, with 2 min of passive rest between trials, and the best time was used for analysis [39].

### Back strength test

The measurements were taken via a Takei (Japan) back and leg dynamometer. Prior to the test, participants were familiarized with the testing procedure through a standardized explanation and practice trial. The participants placed their feet on the dynamometer platform with their knees bent, arms fully extended, back straight, and torso slightly leaning forward. While they gripped the dynamometer bar firmly with their hands, they pulled it upward with maximum effort using their back strength, and the values displayed on the device screen were recorded. The participants were given adequate rest between trials, and the next measurement was taken once they indicated that they were ready. To ensure measurement reliability, each participant performed three trials, and the highest recorded value was used for analysis [40].

### Illinois agility test

The test track was 10 m in length and 5 m in width, with three cones placed in the middle section at 3.3-meter intervals. All test trials were performed on a natural grass surface, and the same surface conditions were maintained for all participants and testing sessions. The measurements were taken via the Smart Speed Lite electronic timing system (Fusion Sport, AU). The track consisted of a 20-meter slalom section, where participants had to make 180-degree turns every 10 m between cones, and a 40-meter straight sprint section. After the Illinois test was set up, an electronic timing system with photocells was placed at the start and finish points, ensuring 0.01-second accuracy. Before the test, the participants were provided with detailed instructions, and the track was introduced. The participants were allowed 3‒4 low-intensity practice trials. Prior to the actual test, the participants performed 5–6 min of warm-up and stretching exercises. During the test, the participants started in a prone position, with their hands touching the ground and aligned with their shoulders. Upon the start signal, they ran through the track, and completion times were recorded in seconds. The test was repeated twice, with full recovery provided between trials. The best recorded time was used for analysis [41].

### Countermovement jump (CMJ) test

The CMJ test was administered to evaluate jump height, which was used as the indicator of lower-body performance in this study. The vertical jump measurements of the participants were conducted via the Smart Speed Lite Smart Jump system (Fusion Sport, AU). Participants stood on the jump mat with their hands fixed firmly on the hips throughout the entire test to eliminate the contribution of arm swing. From this position, they performed a countermovement to approximately 90° of knee flexion before executing a maximal vertical jump. After landing back on the mat, a 1-minute rest interval was provided between attempts. Each participant completed three trials, and the best recorded jump height (cm) was used for analysis [42].

### Statistical analysis

All data were analyzed using SPSS software (version 25.0). The normality of the data was assessed using the Shapiro–Wilk test, and all outcome variables were found to be normally distributed (*p* >.05 for all Shapiro–Wilk tests). For normally distributed data, parametric tests were used. A 2 (Group: EG vs. CON) × 2 (Time: Pre vs. Post) mixed-model repeated measures ANOVA was performed to compare pre- and post-test differences between groups and examine interaction effects. This approach was selected because the study design included only two time points, a balanced dataset with no missing values, and a relatively small sample size, conditions under which repeated-measures ANOVA provides robust and interpretable estimates comparable to linear mixed-effects models. In the absence of missing data or additional hierarchical structure, mixed-effects modelling was not considered necessary. This analytical approach was selected because repeated-measures ANOVA provides greater statistical power and more appropriately models group differences over time compared with independent t-tests on change scores. Since the design included only two measurement points (pre-test and post-test), the assumption of sphericity was inherently met and Mauchly’s test was not applicable. Partial eta squared (ηp²) was reported as the effect size (small: 0.01; medium: 0.06; large: 0.14) [43]. Following a significant Group × Time interaction, post-hoc pairwise comparisons were conducted using paired-samples t-tests (within-group pre- vs. post-test) and independent-samples t-tests (between-group comparisons), with Bonferroni correction applied to adjust for multiple comparisons (adjusted significance threshold: *p* <.0125). The Bonferroni correction was applied only to post-hoc pairwise comparisons because these analyses involve multiple testing and an increased risk of Type I error. In contrast, the main effects and interaction terms derived from the repeated-measures ANOVA represent a priori planned omnibus tests based on the predefined study design and hypotheses; therefore, additional multiplicity correction was not applied to the main ANOVA results, in line with standard statistical practice. The significance level for all other analyses was set at *p* <.05. All randomized participants (*n* = 20) were included in the analysis. No missing data were observed; therefore, no imputation methods were applied. No additional subgroup or sensitivity analyses were performed. All statistical comparisons were conducted based on the predefined primary and secondary outcomes.

## Results

Descriptive statistical results regarding the general characteristics of the youth football players who participated in the study are presented (Table 2).

**Table 2:** General characteristics of the athletes

General Characteristics	EG (*n* = 10)	CON (*n* = 10)
	Mean ± SD	Mean ± SD
Age (years)	12.20 ± 0.79	12.10 ± 0.88
Height (cm)	152.60 ± 9.00	152.70 ± 7.54
Body Weight (kg)	41.07 ± 8.00	40.97 ± 9.36

*Mean* Average, *SD* Standard deviation

The findings of the present study are presented below. To assess the test–retest reliability of the performance measures, intraclass correlation coefficients (ICCs) with 95% confidence intervals (CIs) were calculated (Table 3). The results indicated good-to-excellent reliability across all performance tests, with ICC values ranging from 0.831 to 0.917. The highest reliability was observed in CMJ (ICC = 0.917, 95% CI: 0.91–0.92) and RSI (ICC = 0.907, 95% CI: 0.90–0.92) measures, while the lowest reliability was found in back strength tests (ICC = 0.83, 95% CI: 0.81–0.85). Sprint and COD tests also demonstrated good reliability, with ICCs between 0.84 and 0.89. These findings confirm that the applied performance assessments were stable and reproducible in youth football players.

**Table 3:** Test–retest reliability of performance measures (ICC and 95% CI)

Tests	ICC	95% CI
RSI (pre-test)	0.907	[0.90–0.92]
RSI (post-test)	0.887	[0.87–0.90]
10 m Sprint (pre-test)	0.892	[0.88–0.90]
10 m Sprint (post-test)	0.845	[0.82–0.87]
20 m Sprint (pre-test)	0.865	[0.84–0.88]
20 m Sprint (post-test)	0.889	[0.87–0.90]
30 m Sprint (pre-test)	0.867	[0.84–0.89]
30 m Sprint (post-test)	0.858	[0.83–0.88]
Back Strength (pre-test)	0.831	[0.81–0.85]
Back Strength (post-test)	0.832	[0.81–0.85]
Illinois Agility (pre-test)	0.866	[0.84–0.89]
Illinois Agility (post-test)	0.870	[0.85–0.89]
CMJ (pre-test)	0.917	[0.91–0.92]
CMJ (post-test)	0.894	[0.88–0.90]

ICC values were calculated based on repeated trials in 20 participants (*n* = 20)

*ICC* Intraclass correlation coefficient (measure of test–retest reliability), *95%* *CI* 95% confidence interval for ICC values

Descriptive statistics for the pretest values of the RSI and motor ability tests of the youth football players are presented in Table 4. According to the findings in the table, there was no significant difference between the pretest values of the groups (*p* >.05). These results indicate that the groups were similar in terms of their pretest measurements.

**Table 4:** Repeated-measures ANOVA results for RSI and motor ability outcomes

Variable	Group	Pre-test (Mean ± SD)	Post-test (Mean ± SD)	Δ (Mean ± SD)	Group F(*p*, ηp²)	Time F(*p*, ηp²)	Group × Time F(*p*, ηp²)
RSI (mm/ms)	CON	1.22 ± 0.28	1.27 ± 0.28	+ 0.05 ± 0.09	F(1,18) = 0.335, *p*=.570, ηp²=0.02	F(1,18) = 9.373, *p*=.007, ηp²=0.34	F(1,18) = 4.670, *p*=.044, ηp²=0.21
	EG	1.18 ± 0.25	1.44 ± 0.24	+ 0.26 ± 0.11			
10 m Sprint (sec)	CON	1.98 ± 0.14	1.97 ± 0.11	−0.01 ± 0.12	F(1,18) = 0.278, *p*=.604, ηp²=0.02	F(1,18) = 7.325, *p*=.014, ηp²=0.29	F(1,18) = 4.571, *p*=.046, ηp²=0.20
	EG	2.01 ± 0.13	1.89 ± 0.09	−0.12 ± 0.11			
20 m Sprint (sec)	CON	3.56 ± 0.23	3.55 ± 0.25	−0.01 ± 0.15	F(1,18) = 0.446, *p*=.513, ηp²=0.02	F(1,18) = 8.691, *p*=.009, ηp²=0.33	F(1,18) = 6.529, *p*=.020, ηp²=0.27
	EG	3.57 ± 0.19	3.43 ± 0.15	−0.14 ± 0.12			
30 m Sprint (sec)	CON	5.25 ± 0.32	5.24 ± 0.33	−0.01 ± 0.20	F(1,18) = 0.885, *p*=.359, ηp²=0.05	F(1,18) = 7.242, *p*=.015, ηp²=0.29	F(1,18) = 6.267, *p*=.022, ηp²=0.26
	EG	5.25 ± 0.26	5.03 ± 0.13	−0.22 ± 0.16			
Back Strength (kg)	CON	65.30 ± 11.05	67.05 ± 12.41	+ 1.75 ± 3.10	F(1,18) = 0.088, *p*=.077, ηp²=0.01	F(1,18) = 22.107, *p*<.001, ηp²=0.55	F(1,18) = 5.287, *p*=.034, ηp²=0.23
	EG	65.60 ± 18.03	70.70 ± 17.12	+ 5.10 ± 4.90			
Illinois Agility (sec)	CON	18.50 ± 1.09	18.42 ± 1.20	−0.08 ± 0.18	F(1,18) = 0.024, *p*=.878, ηp²=0.00	F(1,18) = 13.300, *p*=.002, ηp²=0.43	F(1,18) = 7.788, *p*=.012, ηp²=0.30
	EG	18.69 ± 1.13	18.08 ± 1.01	−0.61 ± 0.22			
CMJ (cm)	CON	25.91 ± 4.94	26.30 ± 4.34	+ 0.39 ± 0.71	F(1,18) = 0.002, *p*=.967, ηp²=0.00	F(1,18) = 9.630, *p*=.006, ηp²=0.35	F(1,18) = 6.001, *p*=.025, ηp²=0.25
	EG	24.38 ± 3.36	27.69 ± 3.62	+ 3.31 ± 1.15			

*SD* Standard deviation, *p* Probability value, *ηp²* Partial eta squared (effect size), *Mean* Average, Δ Mean change score, calculated as the difference between post-test and pre-test (Post-test − Pre-test), *F* F-statistic from repeated-measures ANOVA (df = 1,18)

According to the findings in Table 4, no significant difference was found between the pretest values of the groups (*p* >.05). In terms of time, significant differences were detected at a large level in the RSI (*p* =.007; ηp2 = 0.34), 10-m sprint (*p* =.014; ηp^2^ = 0.29), 20-m sprint (*p* =.009; ηp^2^ = 0.33), 30-m sprint (*p* =.015; ηp^2^ = 0.29), back strength (*p* =.000; ηp^2^ = 0.55), COD (*p* =.002; ηp^2^ = 0.43) and CMJ (*p* =.006; ηp^2^ = 0.35) tests. In terms of group and time interaction, large-level significant differences were detected in the RSI (*p* =.044; ηp^2^ = 0.21), 10-m sprint (*p* =.046; ηp^2^ = 0.20), 20-m sprint (*p* =.020; ηp^2^ = 0.27), 30-m sprint (*p* =.022; ηp^2^ = 0.26), back strength (*p* =.034; ηp^2^ = 0.23), COD (*p* =.012; ηp^2^ = 0.30) and CMJ (*p* =.025; ηp^2^ = 0.25) tests. These results indicate that the EG achieved more pronounced improvements in performance outcomes, as reflected by the higher Δ values compared to the CON.

The results of the repeated-measures ANOVA revealed a significant Group × Time interaction, which represents the primary statistical evidence of the intervention’s effectiveness. This interaction demonstrates that the pattern of change over time differed clearly between the groups, with the EG exhibiting greater improvements across all performance outcomes. To further explore this interaction, pairwise comparisons with Bonferroni correction were conducted. The EG demonstrated significant improvements from pre- to post-test in RSI, 20 m sprint, back strength, COD, and CMJ performance (*p* <.0125), with large within-group effect sizes (Cohen’s d = 1.01–3.01), whereas the CON showed no significant changes (Table 5).

**Table 5:** Results of Bonferroni-adjusted pairwise comparisons between groups and time points (t, p, d [95% CI] values)

Variable	EG-CON Pre (t, *p*, d [95% CI])	EG Pre→Post (t, *p*, d [95% CI])	CON Pre→Post (t, *p*, d [95% CI])	EG-CON Post (t, *p*, d [95% CI])
RSI	t = − 0.388, *p* =.703, d = − 0.17 [− 1.05, 0.71]	t = − 3.198, *p* =.011, d = − 1.01 [− 1.77, − 0.22]	t = − 0.780, *p* =.456, d = − 0.25 [− 0.87, 0.39]	t = 1.446, *p* =.166, d = 0.65 [− 0.26, 1.55]
10 m Sprint	t = 0.482, *p* =.636, d = 0.22 [− 0.66, 1.09]	t = 2.867, *p* =.019, d = −0.91 [− 1.64, − 0.17]	t = 0.531, *p* =.608, d = 0.17 [− 0.79, 0.46]	t = −1.760, *p* =.096, d = −0.79 [− 1.70, 0.13]
20 m Sprint	t = 0.083, *p* =.935, d = 0.04 [− 0.84, 0.91]	t = 3.556, *p* =.006, d = −1.12 [− 1.92, − 0.33]	t = 0.310, *p* =.764, d = 0.10 [− 0.72, 0.52]	t = −1.394, *p* =.184, d = −0.62 [− 1.52, 0.28]
30 m Sprint	t = 0.014, *p* =.989, d = 0.01 [− 0.87, 0.88]	t = 2.910, *p* =.017, d = −0.92 [− 1.66, − 0.18]	t = 0.208, *p* =.840, d = 0.07 [− 0.69, 0.55]	t = −1.928, *p* =.078, d = −0.86 [− 1.78, 0.06]
Back Strength	t = 0.045, *p* =.965, d = 0.02 [− 0.86, 0.90]	t = −9.507, *p* <.001, d = 3.01 [1.55, 4.46]	t = −1.292, *p* =.229, d = −0.41 [− 1.05, 0.25]	t = −0.546, *p* =.592, d = −0.24 [− 0.64, 1.12]
Illinois Agility	t = 0.379, *p* =.709, d = 0.17 [− 0.71, 1.05]	t = 4.134, *p* =.003, d = −1.31 [− 2.15, − 0.46]	t = −0.682, *p* =.512, d = −0.22 [− 0.84, 0.41]	t = −0.684, *p* =.503, d = −0.31 [− 1.19, 0.58]
CMJ	t = −0.811, *p* =.429, d = −0.36 [− 1.25, 0.52]	t = −7.667, *p* <.001, d = −2.42 [− 3.67, − 1.15]	t = −0.351, *p* =.734, d = −0.11 [− 0.51, 0.73]	t = 0.775, *p* =.449, d = 0.35 [− 0.54, 1.23]

Statistical significance was set at *p* <.0125 (Bonferroni correction applied)

*t* t-statistic from paired-samples (within-group) or independent-samples (between-group) t-tests, *p* Probability value, *d* Cohen’s d effect size, *95% CI* 95% confidence interval for effect size

According to the post-hoc analysis results presented in Table 5, there were no significant differences between the EG and CON groups at baseline (pre-test), indicating that the groups were comparable at the beginning of the study. For clarity, partial eta squared (ηp²) was used to quantify effect sizes for the repeated-measures ANOVA main and interaction effects, whereas Cohen’s d was calculated to describe the magnitude of within-group pre–post changes. In the EG, significant improvements were observed from pre- to post-test in RSI (d = 1.01), 20 m sprint (d = 1.12), back strength (d = 3.01), COD (d = 1.31), and CMJ (d = 2.42) performance (*p* <.0125), suggesting that the applied intervention had positive effects on physical performance. In contrast, no significant changes were found in the CON group from pre- to post-test in any of the parameters, supporting that the observed improvements were specific to the intervention applied to the EG.

According to the post-test results, no statistically significant differences were found between the EG and CON groups in any parameter (all *p* >.0125). However, these non-significant post-test comparisons should be interpreted as secondary findings and do not contradict the significant Group × Time interaction. Given the strong interaction effect and large within-group improvements in the EG, the results collectively indicate that the HIIT intervention was effective. The lack of between-group significance at post-test likely reflects the study’s limited statistical power due to the relatively small sample size (*n* = 10 per group) and high inter-individual variability, which increased standard deviations and reduced sensitivity to detect between-group mean differences.

**Fig. 3:**
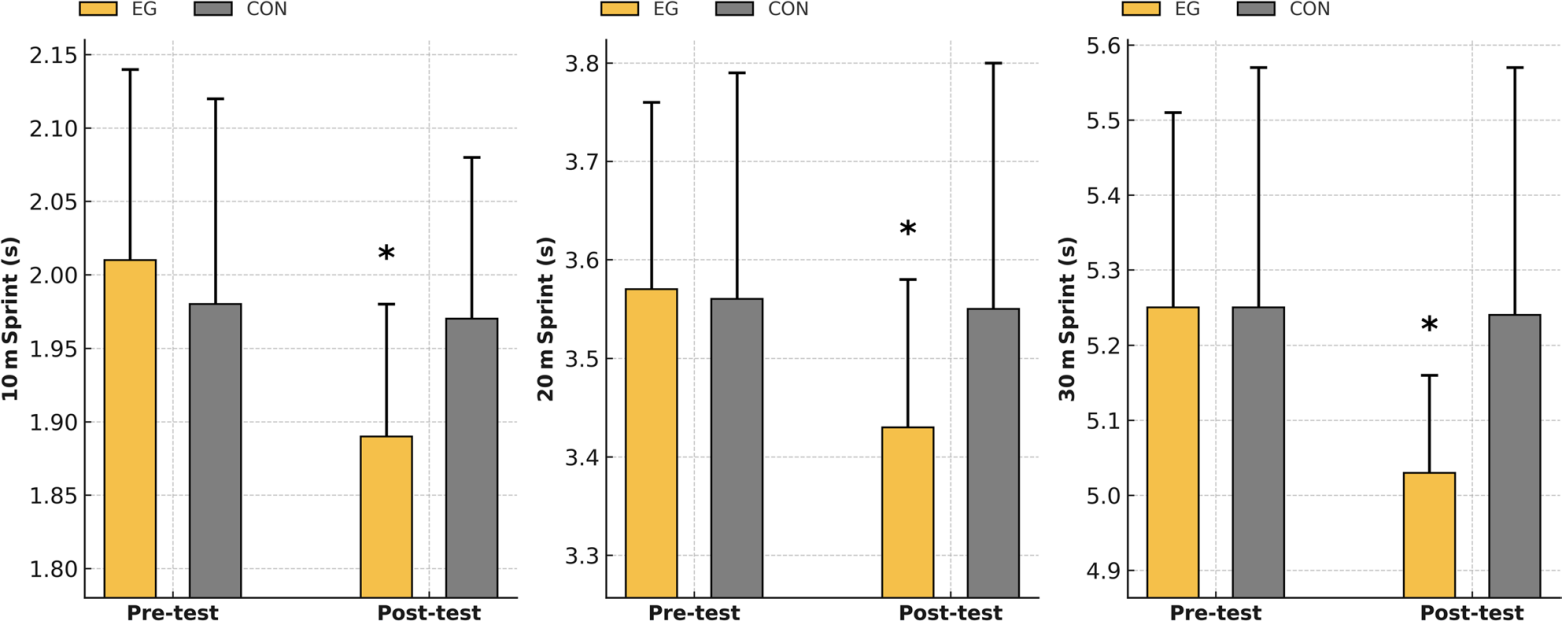
Changes in 10-, 20-, and 30-m sprint performance before and after the intervention in EG and CON. Error bars represent standard deviation (SD). The Group × Time interaction was significant, indicating a different rate of improvement between groups

The sprint performance findings of the youth football players are presented graphically (Fig. 3). As shown in Fig. 3, the EG demonstrated clear improvements in 10-m, 20-m, and 30-m sprint times, while the CON group showed minimal change. In terms of sprint performance, significant improvements were observed in the EG following the HIIT intervention. These results indicate that the HIIT program effectively enhanced short-distance sprint ability, particularly acceleration and maximal sprinting speed, which are critically important components of football performance.

**Fig. 4:**
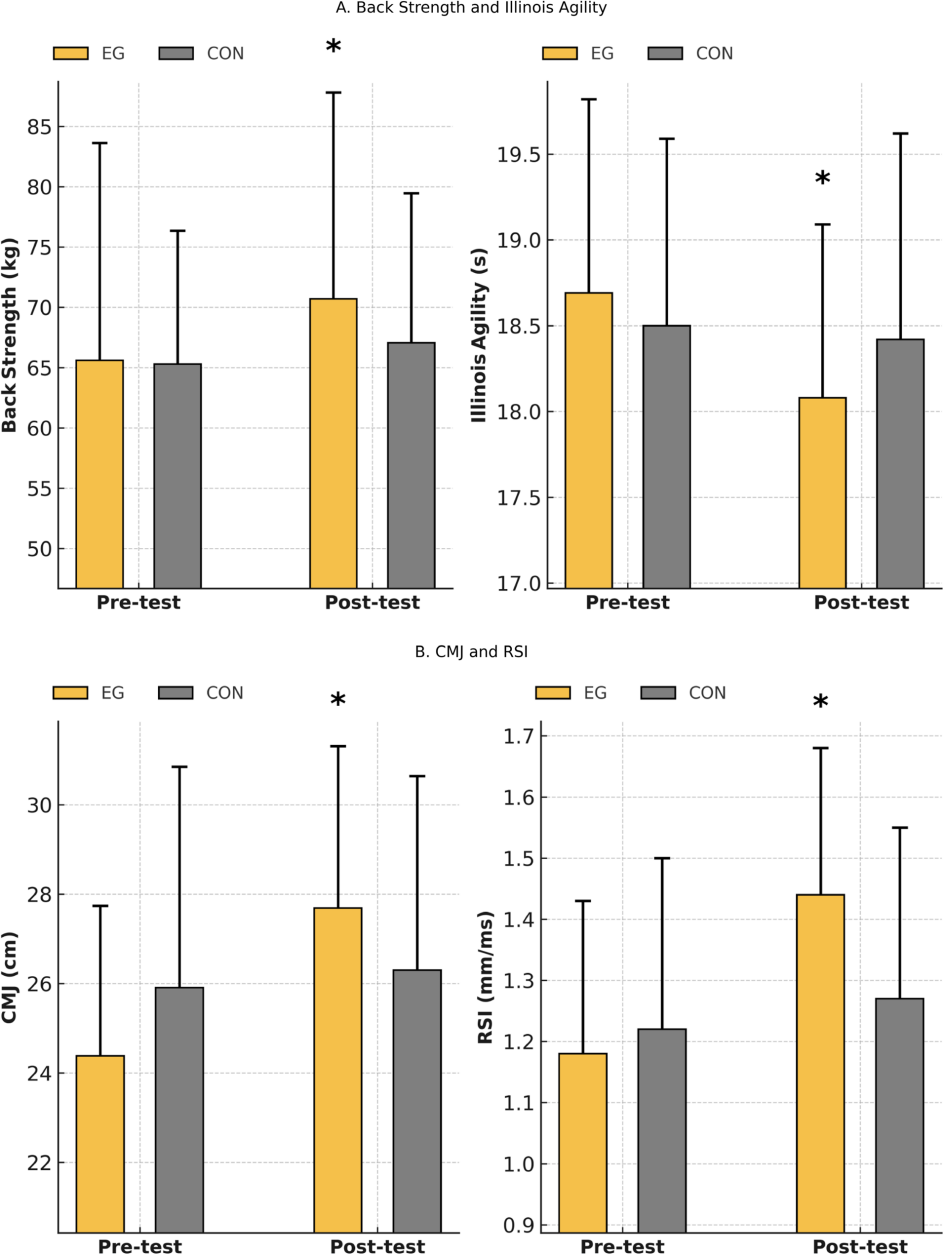
Changes in back strength, Illinois agility performance, countermovement jump (CMJ), and reactive strength index (RSI) before and after the intervention in the experimental group (EG) and control group (CON). Panel **A** illustrates changes in back strength and Illinois agility, while Panel **B** presents changes in CMJ and RSI. Error bars represent standard deviation (SD). A significant Group × Time interaction was detected for all variables, indicating that performance changes over time differed between the groups

The findings related to back strength, COD, CMJ, and RSI performance are presented graphically (Fig. 4). As shown in Fig. 4, the EG demonstrated clear post-intervention improvements across all performance variables following the HIIT intervention, whereas the CON exhibited no change. Specifically, marked enhancements were observed in back strength and Illinois Agility Test performance, indicating meaningful gains in trunk muscle strength and COD ability, both of which are essential components of football performance. In addition, substantial improvements in CMJ height and RSI values were evident in the EG after the intervention, reflecting enhanced lower-limb explosive power and stretch–shortening cycle efficiency. Collectively, these findings indicate that the HIIT program elicited broad neuromuscular adaptations that translated into improved strength, agility, and explosive performance capacities in youth football players.

## Discussion

This study aimed to determine the effects of a HIIT intervention on RSI, sprint performance, back strength, COD, and CMJ in youth football players aged 11–13 years. The repeated-measures ANOVA revealed significant main effects of time and a significant group × time interaction, indicating that both the rate and magnitude of improvement differed between groups. The EG exhibited substantial and statistically significant improvements across all performance variables, including RSI, sprint (10 m, 20 m, and 30 m), back strength, COD, and CMJ, while the CON group showed minimal or no changes. The large within-group effect sizes observed in the EG further strengthen the evidence supporting the effectiveness of incorporating HIIT into regular football training. Although the post-test group means did not differ significantly, this outcome is most likely attributable to high inter-individual variability and the limited statistical power associated with the small sample size rather than a lack of intervention effect. Importantly, the absence of statistically significant between-group differences at post-test does not undermine the effectiveness of the intervention, as it was supported by significant Group × Time interactions and large within-group performance gains in the EG. Therefore, between-group post-test comparisons should be interpreted with caution, and future studies with larger samples are warranted to confirm these findings. Overall, these findings demonstrate that HIIT elicited meaningful and practically relevant performance enhancements that were not achieved through regular training alone. However, it should be acknowledged that although total session duration was matched between groups, the HIIT-based intervention imposed a higher physiological and neuromuscular stimulus than the low-to-moderate-intensity technical–tactical activities performed by the CON, which should be considered a potential confounding factor when interpreting the comparative effects of the intervention. Moreover, the absence of objective internal load monitoring may have influenced the interpretation of motor performance adaptations, as potential mismatches in physiological load between groups could bias responsiveness in outcomes such as sprint performance, COD, and RSI. In addition, given that the studied age range (11–13 years) coincides with peak height velocity and rapid neuromuscular maturation, inter-individual differences in biological maturation may have influenced training responsiveness and should be considered when interpreting the present findings.

RSI is widely recognized as a key indicator of explosive strength and stretch–shortening cycle efficiency, and it has been strongly linked to sprinting, jumping, and change of direction performance in football players [11, 12]. According to the results of our study, HIIT led to significant improvements in RSI (*p* =.044; ηp² = 0.21) in the EG, while no changes were observed in the CON. These findings align with previous research demonstrating that improvements in strength, speed, and CMJ performance are closely associated with increases in RSI [44]. The most likely explanation is that enhanced neuromuscular adaptations resulting from HIIT—such as increased motor unit recruitment and synchronization, improvements in rate of force development (RFD), and greater tendon stiffness enabling more efficient elastic energy storage and release—contribute to better reactive strength performance. From a practical football perspective, these adaptations may translate into faster acceleration, more explosive take-offs, and improved efficiency during rapid directional changes. Similarly, HIIT interventions applied to U19 female football players resulted in significant improvements in RSI at drop jump (DJ) 30 cm (*p* =.019) and DJ 40 cm (*p* =.026) [45]. These consistent outcomes across different age groups suggest that HIIT may effectively stimulate the stretch–shortening cycle through repeated high-intensity running efforts, which mirror the rapid eccentric–concentric actions required in reactive jumping and sprinting. The ability of HIIT to induce both central (cardiorespiratory) and peripheral (neuromuscular) adaptations likely underlies these performance enhancements. Collectively, these findings indicate that HIIT can effectively enhance reactive strength and explosive performance in youth football players, providing coaches with an efficient and time-effective training strategy to improve lower-limb power and dynamic performance capacity within regular football training schedules.

Sprinting ability is one of the most critical motor skills in football, as it directly influences decisive actions such as creating goal-scoring opportunities and defensive recovery runs [5]. In our study, no improvement in sprint performance was observed in the CON, whereas the EG demonstrated significant gains in both acceleration and maximal sprinting speed (10 m: *p* =.046, ηp² = 0.20; 20 m: *p* =.020, ηp² = 0.27; 30 m: *p* =.022, ηp² = 0.26). These results show that HIIT effectively improves different phases of sprint performance. This finding aligns with the existing literature indicating that HIIT can produce rapid and meaningful enhancements in sprint ability within relatively short training periods [46]. García-Pinillos et al. [47] reported significant sprint improvements and a meaningful group × time interaction following a 5-week running-based HIIT program, while Gökkurt and Kıvrak [48] observed notable gains in speed and acceleration after an 8-week HIIT protocol with U19 football players. Similarly, Wong et al. [49] showed that combining muscular strength training with HIIT improved 10-m and 30-m sprint times in professional players. Michailidis et al. [31] also demonstrated significant 10-m sprint improvements after a 4-week HIIT intervention in U17 footballers. Stanković et al. [50] found that both linear and change-of-direction HIIT protocols enhanced sprint performance in elite female players, although neither method produced superior outcomes. Overall, previous research supports the substantial improvements observed in the present study, confirming the effectiveness of HIIT in enhancing sprint-related physical qualities in youth football players.

Strength is widely recognized as a key determinant of physical performance in football, as it directly influences crucial actions such as duels, jumping and heading, sprinting, and other explosive movements [51]. In the present study, while no improvement was observed in the CON, the EG showed significant increases in back strength (*p* =.034; ηp² = 0.23). Although HIIT is primarily considered a running-based metabolic stimulus, the notable improvement in back strength represents an unexpected yet meaningful finding that warrants deeper interpretation. Nevertheless, this finding should be considered exploratory in nature, as direct measures of trunk muscle activation were not included in the present study. Accordingly, the underlying mechanisms remain speculative and should be interpreted with caution. A plausible explanation may be that repeated high-intensity running efforts require substantial trunk and posterior chain stabilization to maintain optimal sprint posture. During maximal acceleration and upright sprinting, the erector spinae, multifidus, gluteal muscles, and deep core stabilizers must generate high levels of isometric and dynamic force to ensure effective force transmission through the kinetic chain [52, 53]. Sustained exposure to these demands may enhance neuromuscular coordination between trunk stabilizers and the lower limbs, improving force transfer efficiency and allowing greater force expression during maximal back strength testing [54, 55]. Furthermore, the acceleration–deceleration demands inherent to HIIT involve repeated eccentric braking and postural control, which may increase loading on the posterior kinetic chain and contribute to enhanced trunk stability and greater neuromuscular activation of the spinal extensor musculature [56, 57]. Over time, these repeated eccentric–isometric loading patterns may promote structural and neural adaptations, such as increased muscle activation capacity, improved intermuscular coordination, and enhanced stiffness of trunk-related connective tissues, all of which are relevant to maximal force production [58]. Nevertheless, these proposed mechanisms should be viewed as theoretical interpretations rather than definitive explanations, given that trunk muscle activation and internal load were not directly measured in the present study. This finding is consistent with previous research demonstrating that HIIT can enhance strength-related performance in football players. Chmura et al. [59] reported that a four-week HIIT program improved overall physical performance and load tolerance in young football players. Similarly, Buchheit and Laursen [19] highlighted that repeated high-intensity efforts inherent to HIIT stimulate muscular force production and contribute to improved strength and power output in team-sport athletes. Singh et al. [60] also observed that interval training led to significant gains in muscular strength and other motor abilities among youth football players. From a functional perspective, improved trunk and posterior chain strength may also support sprint mechanics, postural stability, and force application during football-specific actions, thereby reinforcing the practical relevance of the observed back strength adaptations. Taken together, while the present results suggest that running-based HIIT may positively influence back strength, this outcome should be interpreted as exploratory and warrants confirmation in future studies incorporating direct assessments of trunk muscle activation and strength.

HIIT is considered highly important in team sports because it provides rapid and practical improvements in key performance parameters such as COD [46, 61]. For this reason, COD should be integrated into training programs in diverse and complementary ways [62]. In the present study, while no improvement in COD was observed in the CON, the EG demonstrated significant enhancements in COD performance (*p* =.012; ηp^2^ = 0.30), highlighting the positive impact of the HIIT intervention. Kayhan et al. [8] reported that RSI is significantly correlated with COD performance in young football players, suggesting that RSI should be interpreted in relation to strength and COD abilities. These findings support the potential of HIIT to enhance COD performance in youth football players. In elite female players, a direct comparison of linear vs. COD-based HIIT produced significant within-group COD and sprint gains in both arms without a differential group × time effect (ANCOVA *p* >.05) [51], aligning with our observation that improvements can be substantial within groups even when post-test means between groups are statistically indistinguishable. Similarly, a study reported that a HIIT intervention applied to U15 football players led to improvements in COD performance [63]. Previous research has also revealed that HIIT exercises have a significant effect on COD performance (*p* =.000), indicating a strong influence of high-intensity interval training on agility and direction-change ability in football players [64]. One study examined the effects of HIIT combined with Tabata training on COD development and reported that both training models effectively improved COD performance, with statistically significant results in both protocols (*p* =.000) [65]. Supporting these findings, an eight-week periodized interval training program combined with explosive strength and speed significantly improved COD performance (*p* =.000), as measured by the Arrowhead Agility Test, in youth football players [66]. In female youth football players, an eight-week HIIT intervention resulted in greater improvements in COD performance compared with resistance training [67]. Moreover, a four-week HIIT program focusing on sport-specific techniques was shown to enhance jumping ability and COD speed in young athletes [14].

CMJ performance is widely recognized as a key indicator of athletic ability in football, reflecting lower-limb explosive strength and showing strong associations with short-distance sprint performance [68]. In the present study, while no improvement was observed in the CON, the EG demonstrated significant increases in CMJ performance (*p* =.025; ηp² = 0.25). These findings align with previous studies reporting that high-intensity training can enhance jumping ability in football players. Nayıroğlu et al. [45] observed significant improvements in CMJ performance following an eight-week running-based HIIT program in U19 female players. Similarly, Wong et al. [49] reported that a preseason program combining muscular strength training with HIIT led to marked improvements in vertical jump performance among professional football players. Wen et al. [69] also found that HIIT improved CMJ outcomes in youth players, albeit to a lesser extent than alternative methods. Beyond general increases in explosive strength, several physiological mechanisms may explain the CMJ improvements observed in this study. HIIT likely enhanced RFD, enabling athletes to produce higher propulsive forces during the concentric phase of the jump. Additionally, repeated high-intensity efforts may have increased motor unit recruitment and improved tendon stiffness, allowing for more efficient elastic energy storage and release, ultimately contributing to greater jump height. Overall, the present findings reinforce the evidence that HIIT is an effective strategy to enhance lower-limb power and vertical jump performance in football.

When interpreting the present findings in relation to previous studies, several similarities and differences should be considered. While prior HIIT studies in football players have generally reported improvements in sprinting, jumping, and COD performance, many of these investigations involved older adolescents or adult players, longer intervention durations, or combined strength–conditioning programs. In contrast, the present study focused specifically on early adolescent male football players (11–13 years) and applied a short-term, running-based HIIT protocol integrated into regular football training. These differences in sample characteristics, training content, and intervention duration may partly explain variations in the magnitude of adaptations reported across studies. Moreover, whereas some studies quantified external or internal training load using objective measures, the present study relied on a time-matched design without direct load monitoring, which should be considered when comparing outcomes. Despite these methodological differences, the overall pattern of within-group improvements observed in the EG is consistent with the existing literature, supporting the efficacy of HIIT for enhancing key performance parameters in youth football players.

From an applied perspective, the findings of this study suggest that a short-duration, running-based HIIT block can be feasibly integrated into youth football training to enhance neuromuscular and motor performance. Coaches may consider implementing HIIT sessions three times per week over a 4-week period, by replacing a low-to-moderate-intensity technical–tactical segment rather than adding extra training volume. When appropriately designed, such HIIT blocks may contribute to improvements in sprint performance, COD ability, RSI, and lower-body power without substantially increasing overall session duration. Given the young age of the athletes, careful attention should be paid to exercise intensity, recovery intervals, and technical execution to ensure training safety and minimize injury risk. When applied progressively and within an age-appropriate framework, short-term HIIT interventions may represent an effective and time-efficient strategy for enhancing performance in youth football players.

## Conclusions

This study examined the effects of HIIT on RSI, sprint, back strength, COD, and CMJ performance in youth football players aged 11–13 years. According to the findings, no significant changes were observed in the CON, whereas the EG showed significant improvements across all performance parameters. However, between-group comparisons at post-test did not reach statistical significance, despite the favorable within-group changes observed in the EG. This pattern should be interpreted cautiously, as the study was underpowered for detecting between-group post-test differences, increasing the risk of Type II error (i.e., failing to detect a true between-group effect). These results support the idea that HIIT may be considered an important method for the development of young football players within the context of the present sample, as it leads to significant progress in sprint, strength, and COD key performance parameters in football and can therefore serve as a complementary strategy to traditional training methods. Nevertheless, conclusions regarding superiority over traditional training should be drawn with caution given the non-significant post-test between-group differences and limited statistical power. Accordingly, future studies with larger samples are needed to confirm the robustness and generalisability of these findings. Therefore, HIIT can be considered an effective training method for enhancing both physical performance and overall health in young athletes when applied to similar youth football settings.

### Limitations

This study has certain limitations. The fact that the participants were football players aged 11–13 introduces uncertainty regarding whether similar results would be observed across different age groups. Additionally, the lack of follow-up prevented an evaluation of whether the effects of HIIT interventions were sustained beyond the intervention period. Therefore, the short intervention duration (4 weeks) and the absence of a follow-up assessment limit conclusions regarding the long-term retention of the observed adaptations. Another important factor is that, while participants and their families were instructed to maintain proper nutrition and sleep habits, there was no absolute control over these factors, which could influence performance outcomes. Moreover, potential confounding factors such as maturation status and training history were not controlled, and no objective maturation indicator (e.g., peak height velocity) was assessed, which could have influenced the observed results. Given that the studied age range (11–13 years) coincides with peak height velocity and rapid neuromuscular development, inter-individual differences in biological maturation may have influenced training responsiveness and limit the generalisability of the findings. Accordingly, the observed adaptations should be interpreted with caution, and future studies should incorporate objective maturation assessments. Furthermore, because the HIIT block replaced a low-to-moderate-intensity technical–tactical segment rather than adding extra training time, the two groups were exposed to fundamentally different training stimuli. As a result, the EG experienced a substantially higher physiological and neuromuscular load compared to the CON, whose activities consisted primarily of lower-intensity technical–tactical drills. This difference in neuromuscular and metabolic demand is inherent to HIIT interventions but may have partially contributed to the observed improvements; therefore, the findings should be interpreted with consideration of this stimulus-related neuromuscular load discrepancy. In addition, internal training load was not directly monitored using validated methods such as session rating of perceived exertion (sRPE) or heart rate–based training impulse (TRIMP). The absence of objective internal load quantification limits the ability to determine whether the physiological stress imposed by the intervention was equivalent between groups and should be considered when comparing the magnitude of adaptations. A further limitation is that no significant between-group differences were found at the post-test, despite large within-group improvements in the EG. Our a priori power analysis was based on a large effect size (f = 0.40) for the Group × Time interaction. While this interaction was detected, the study was likely underpowered to detect significant between-group differences in post-test means, as assessed by independent t-tests, given the observed variability and sample size (*n* = 10 per group). This reduced sensitivity may explain the absence of post-test group differences, even though large within-group effect sizes (Cohen’s d) were observed, providing strong evidence of the intervention’s effectiveness. Another limitation of the current study lies in the estimation of the maximum heart rate (HR_max); therefore, the calculated target heart rate zones should be interpreted with caution. Finally, the effects of the applied training protocol in terms of individual differences and players’ game positions were not assessed. Additionally, the study sample consisted exclusively of male players recruited from a single football academy on a voluntary basis. This sampling approach may introduce selection bias and limits the generalizability of the findings to female athletes, players of different age groups, or those competing at other performance levels or training environments. Overall, despite the presence of a significant Group × Time interaction, the study was underpowered to detect between-group differences in the post-test phase, and this limitation must be considered when interpreting the comparative outcomes. Accordingly, the mismatch in internal physiological and neuromuscular load between the HIIT-based intervention and the low-to-moderate-intensity technical–tactical activities performed by the CON should be considered a potential confounding factor when interpreting the comparative effects of the intervention.

### Recommendations

Future studies should examine the effects of similar training protocols on football players of different age groups and skill levels. In addition, future research should include female football players to improve the generalizability of findings across sex and competitive contexts. The long-term effects of HIIT in youth populations remain unclear, and future longitudinal studies with follow-up assessments are needed to determine whether the improvements observed in the present study can be sustained beyond the intervention period. Comprehensive research that considers factors such as nutrition, sleep, and individual physiological differences could provide a more detailed understanding of the impact of HIIT on sports performance. Furthermore, the effectiveness of position-specific HIIT protocols for players in different football positions should be investigated. Given the influence of biological maturation on training responsiveness during adolescence, future studies are encouraged to integrate objective maturation assessments, such as peak height velocity (PHV) or Tanner staging, when examining training adaptations in youth football players. In particular, stratifying participants according to maturation status may help clarify dose–response variability to HIIT during this critical neuromuscular development window. Future studies are also encouraged to incorporate maximal graded exercise testing where possible to improve precision in training zone determination. In addition, future research should include internal load measurements such as sRPE to better quantify and match the training stimulus between groups. Finally, future research may explore the effectiveness of hybrid training programs, where HIIT is integrated with traditional training methods, including tactical periodization approaches commonly used in football training. Such hybrid designs may better reflect real-world training environments and enhance the ecological validity of intervention-based research in football.

## Supplementary Information

Supplementary Material 1.

Supplementary Material 2.

## Data Availability

The datasets used and analyzed during the current study are not publicly available due to ethical restrictions related to child participants but are available from the corresponding author upon reasonable request.

## Abbreviations

SPSS: Statistical Package for the Social Sciences
%: Percentage
N: Number of Participants
P: Significance Level
ηp^2^: Partial Eta Squared
CMJ: Countermovement Jump
RSI: Reactive Strength Index
COD: Change of Direction
THR: Target Heart Rate
RFD: Rate of Force Development
sRPE: Session Rating of Perceived Exertion
TRIMP: Heart Rate–Based Training Impulse
PHV: Peak Height Velocity
HIIT: High-Intensity Interval Training
