# Supplementary material for: High-intensity interval training improves the reactive strength index and motor ability of youth football players
Source: BMC Sports Sci Med Rehabil. 2026 Feb 2;18:137. doi: 10.1186/s13102-026-01560-9 (PMC13001278; doi:10.1186/s13102-026-01560-9)
Supplement: Supplementary file 1 — Supplementary Material 1. [file 13102_2026_1560_MOESM1_ESM.docx]

**Appendix 1. Adapted CERT Checklist – Description of the HIIT Intervention**

| **CERT Item** | **Description** |
| --- | --- |
| **1. Nature of the training program** | A structured high-intensity interval training (HIIT) protocol was implemented in addition to regular football training to enhance explosive strength, agility, and speed in 11–13-year-old male football players. |
| **2. Specific exercises performed** | Each session included 2 sets of 6 repetitions of 15-second sprints performed at 80–90% of maximal intensity, with 30 seconds of rest between repetitions and 3 minutes between sets. A standardized 15-minute warm-up (slow running, stretching, jumping drills) preceded each session. |
| **3. Format of delivery (individual/group)** | Training was delivered in small groups (n=10, experimental group), but each athlete performed the sprints individually under supervision. |
| **4. Training location and setting** | All sessions took place on a natural grass football field under controlled weather and environmental conditions, prior to regular football practice. |
| **5. Supervision and instructor qualifications** | Sessions were supervised by sports science researchers and licensed coaches with HIIT training experience to ensure safety and protocol compliance. |
| **6. Training frequency and schedule** | The program lasted 4 weeks, with 3 sessions per week (Monday, Wednesday, Friday), totaling 12 sessions. |
| **7. Structure of a typical session** | Each session included: (1) 15-minute warm-up (7 min jogging, 4 min stretching, 4 min jumping), (2) ~8 minutes of HIIT work, (3) cooldown and recovery. |
| **8. Intensity and exertion level** | Training intensity progressed weekly from 80% to 90% of estimated HRmax. Intensity was verified using heart rate calculations (Karvonen method). Participants operated at high-intensity effort (RPE 7–9). |
| **9. Duration and volume of training** | Each sprint lasted 15 seconds, and total HIIT time per session was approximately 7.5 minutes, excluding warm-up and cooldown. |
| **10. Progression model** | Weekly progression was planned by increasing sprint intensity and maintaining set volume. Heart rate monitoring ensured participants reached the target zone each week. |
| **11. Individual adaptation** | All participants followed the same protocol; however, rest was extended when signs of fatigue were observed. No participant was excluded for underperformance. |
| **12. Adherence and compliance** | Attendance was recorded at each session. Participants were expected to attend all 12 sessions, and full adherence (12/12 sessions) was achieved by all participants in the experimental group. |
| **13. Co-interventions or additional training** | The control group continued their routine football training only. The experimental group performed HIIT before regular football practice. No additional structured training was allowed. |
| **14. Feedback and motivation strategies** | Real-time verbal encouragement was provided during training. Participation was tracked, and athletes were informed of their progress. |
| **15. Safety and adverse events** | All sessions included structured warm-up and cooldown periods. All training was conducted under supervision. No adverse events were reported. |
| **16. Outcome measures and evaluation** | RSI, CMJ, sprint, agility, and back strength were measured using a pretest–posttest design by blinded assessors using standardized protocols. |
